# Alternative Extraction and Characterization of Nitrogen-Containing Azaphilone Red Pigments and Ergosterol Derivatives from the Marine-Derived Fungal *Talaromyces* sp. 30570 Strain with Industrial Relevance

**DOI:** 10.3390/microorganisms8121920

**Published:** 2020-12-03

**Authors:** Juliana Lebeau, Thomas Petit, Mireille Fouillaud, Laurent Dufossé, Yanis Caro

**Affiliations:** 1 Laboratoire de Chimie et de Biotechnologie des Produits Naturels, CHEMBIOPRO, Université de La Réunion, 15 Avenue René Cassin, CS 92003, F-97744 Saint-Denis, France; juliana.lebeau@univ-reunion.fr (J.L.); thomas.petit@univ-reunion.fr (T.P.); mireille.fouillaud@univ-reunion.fr (M.F.); laurent.dufosse@univ-reunion.fr (L.D.); 2 Département Hygiène Sécurité Environnement (HSE), IUT La Réunion, Université de La Réunion, 40 Avenue de Soweto, BP 373, F-97455 Saint-Pierre, France

**Keywords:** *Talaromyces*, azaphilone, marine fungi, *N*-threonyl-rubropunctamin, PP-R, greener extraction, red pigments, fungal pigments

## Abstract

Many species of *Talaromyces* of marine origin could be considered as non-toxigenic fungal cell factory. Some strains could produce water-soluble active biopigments in submerged cultures. These fungal pigments are of interest due to their applications in the design of new pharmaceutical products. In this study, the azaphilone red pigments and ergosterol derivatives produced by a wild type of *Talaromyces* sp. 30570 (CBS 206.89 B) marine-derived fungal strain with industrial relevance were described. The strain was isolated from the coral reef of the Réunion island. An alternative extraction of the fungal pigments using high pressure with eco-friendly solvents was studied. Twelve different red pigments were detected, including two pigmented ergosterol derivatives. Nine metabolites were identified using HPLC-PDA-ESI/MS as *Monascus*-like azaphilone pigments. In particular, derivatives of nitrogen-containing azaphilone red pigment, like PP-R, 6-[(Z)-2-Carboxyvinyl]-*N*-GABA-PP-V, *N*-threonine-monascorubramin, *N*-glutaryl-rubropunctamin, monascorubramin, and presumed *N*-threonyl-rubropunctamin (or acid form of the pigment PP-R) were the major pigmented compounds produced. Interestingly, the bioproduction of these red pigments occurred only when complex organic nitrogen sources were present in the culture medium. These findings are important for the field of the selective production of *Monascus*-like azaphilone red pigments for the industries.

## 1. Introduction

With the progress of biotechnologies, the investigation and exploitation of rich natural sources to isolate natural products with commercial applications has gained increasing interest. Interestingly, the quest for novel drugs has driven research back to look closer at what nature has to offer: biodiversity and untapped natural resources [1,2]. Microorganisms represent a vast repertoire of natural products, many of them with industrial importance. Industrially important fungal bioactive compounds, such as enzymes, organic acids, biochemicals and pigments (with shades of orange, yellow, red, etc.), can be produced from specific fungi [3,4,5,6,7]. As some synthetic colorants have carcinogenic and teratogenic effects, fungal pigments represent an alternative source of natural colorants that are independent of agro-climatic conditions [7,8]. Red colorants of fungal origin have become more and more valued and sought after in the industries, like textiles, food, cosmetics, and pharmaceutics [9,10]. Indeed, to this day, very few stable red colorants of natural origin are available for the industries. Consequently, fungal red pigments are now well established in the industry among the natural colorants, competing with plant and microalgae pigments [3,11].

Fungi of marine origin represent a source of active metabolites exerting pharmacological properties for drug applications [2,6]. In accordance with their genetic potential, some marine-derived fungal strains of *Talaromyces* produced toxin-free polyketide-based pigments and could then be exploited in the industries as a non-toxigenic fungal cell factory in future. Polyketide-based pigments are characterized by a multitude of complex and diverse chemical structures, including quinones (naphthoquinones, hydroxy-anthraquinones) and azaphilones [5]. They involve biosynthetic pathways catalyzed by multiple polyketide synthase enzymes (PKS). The biological properties of fungal azaphilone pigments with pyrone–quinone structures may open new avenues for their use in the production of valuable drugs for medical use. Since ancient times, the fermentation of *Monascus* species has been used to color food products (like meat, wine, cheese, rice and koji) in Asian far-east countries. These fungi produced well-known, yellow-orange-red, azaphilone-based pigments [3,5], but their use as food colorants is not allowed in the USA and in European countries due to the occasional occurrence of the mycotoxin citrinin, along with the undesirable compound mevinolin [9,12,13,14,15]. Recent studies have shown that some *Talaromyces/Penicillium* sp. non-pathogenic to humans, such as *Talaromyces aculeatus*, *T. pinophilus*, *T. funiculosus*, *T. atroroseus*, *T. minioluteus*, *T. marneffei* and *T. albobiverticillius*, naturally secrete soluble *Monascus*-like azaphilone red pigments and their amino acid derivatives, without side-production of mycotoxins [15,16,17,18]. *Talaromyces/Penicillium* species are promising sources of fungal polyketide-based red pigments (monascorubramin, rubropunctamin, PP-R, etc.), which can be safely applied in the industries (such as animal feed supplementation, foods, nutraceuticals, pharmaceuticals and cosmetics) [19]. More recently, studies performed by Chen et al. [20,21] and Liu et al. [22] have explained the biosynthetic pathway of *Monascus*-like azaphilone pigments in *Monascus* and *Talaromyces/Penicillium* genera. They described a common gene cluster responsible for the pigment production in these genera, as well as differences regarding the gene organization, copy numbers and allelic diversity.

Traditionally, the *Monascus*-like azaphilone pigments are being extracted from microbial biomass by conventional solid–liquid extraction processes and require extended extraction times, high temperatures and important volumes of various organic solvents (*n*-hexane, acetone, chloroform, ethyl acetate, etc.) [23]. Extracting the fungal pigments via green processing technologies presents a promising approach to pursue a more sustainable production of natural colorants [24]. Therefore, alternatives are assessed for pigment extraction by different technologies (e.g., extraction assisted by ultrasound, microwave, enzymatic or high-pressure treatments) [5,25,26,27].

The aim of this study is focused on the characterization of the target bioactive compounds (e.g., derivatives of nitrogen-containing azaphilone red pigments and ergosterol derivatives) produced by a wild type of *Talaromyces* sp. 30570 (CBS 206.89 B) marine-derived fungal strain isolated from the coral reef of the Réunion island. The influence of the nutrients’ profile on the fungal pigments production in two submerged cultures, either with simple or complex sources of nitrogen, were also studied. Furthermore, we investigated the use of an alternative Pressurized Liquid Extraction (PLE) method based on the extraction procedure published in our previous work [25], using eco-friendly solvents (e.g., water, methanol and/or ethanol, which are allowed in the US and in the EU for the extraction of natural products), for advanced mycelial pigment extraction from the marine-derived *Talaromyces* sp. 30570 fungal strain. This alternative extraction consists of a high-pressure extraction process from the mycelial cells carried out at a high temperature and elevated pressure (>10 MPa) in order to maintain the solvents at liquid state when applied to the sample, as well as to maximize the extraction efficiency [5,25]. The pigment composition was characterized by high-performance liquid chromatography-diode array detection-electrospray ionization mass spectrometry (HPLC-PDA-ESI/MS).

## 2. Materials and Methods

### 2.1. Submerged Fermentation of Fungal Strain

**Figure 1:**
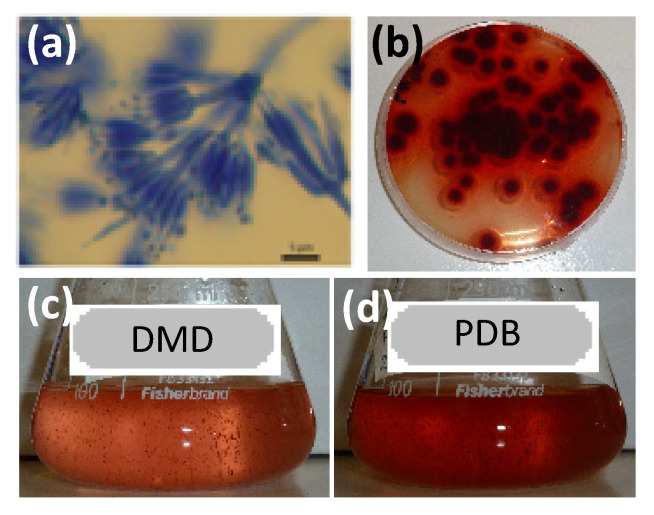
Morphological features of the marine-derived *Talaromyces* sp. 30570 strain: (**a**) Conidiophores produced on Potato Dextrose Agar (PDA) media, stained with lactophenol blue (scale bar 5 μm); (**b**) Reverse face of fungus grown on PDA; (**c**) Red pigment production in Defined Minimal Dextrose (DMD) medium incubated for 7 days at 24 °C; (**d**) Red pigment production in Potato Dextrose Broth (PDB) medium incubated for 7 days at 24 °C.

The fungal strain was sampled from Réunion island coral-reef according to our previous study and was identified as *Talaromyces* sp. CBS 206.89 B (collection strain No. 30570 in our collection reference system) using morphological observation and gene sequencing [28]. Two submerged culture media, the Defined Minimal Dextrose broth (DMD) and the Potato Dextrose Broth (PDB), containing simple source of nitrogen (i.e., ammonium sulfate) and complex sources of nitrogen (like amino acids and proteins), respectively, were used for the comparison in terms of pigment production in submerged fungal cultures, as reported earlier [25]. Culture pH medium was adjusted to 6.0 ± 0.2 prior to sterilization. Pre-culture was prepared by taking a loop of fungus from 7-day old culture grown on PDA Petri plates and transferred into 60 mL sterilized PDB culture medium. The flasks were incubated at 26 °C for 72 h. Cultures were carried out in 250 mL Erlenmeyer flasks containing 100 mL of sterilized culture medium. The flasks were inoculated with 1% (*w/v*) 72 h-old seed culture and incubated at 26 °C for 7 days at 150 rpm (Infors Multitron HT) (Figure 1).

### 2.2. Biomass Separation, Extraction and Quantification of the Polyketide-Based Pigments

**Figure 2:**
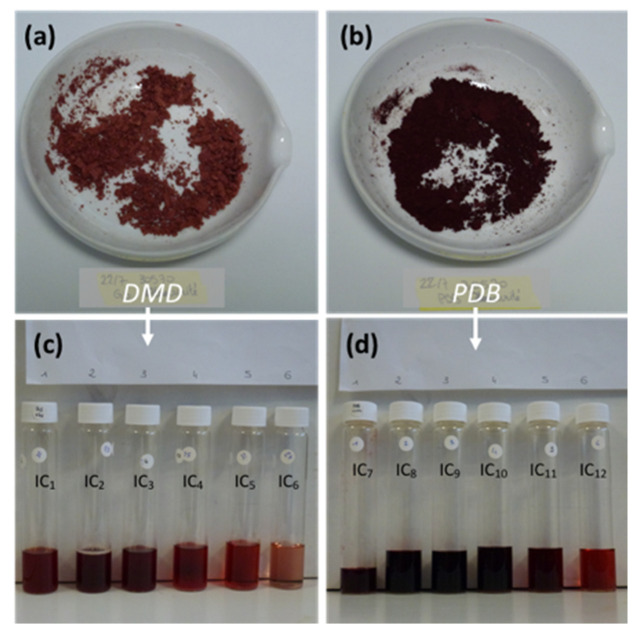
Red-colored dried residues obtained from submerged culture of the marine-derived *Talaromyces* sp. 30570 strain (**a**) in DMD, or (**b**) in PDB medium; (**c**,**d**) intracellular (IC) liquid extracts obtained using the pressurized liquid extraction method on mycelial cells of *Talaromyces* sp. 30570. The sequence of solvents was set to display a decreasing polarity profile (from IC_1_ to IC_6_; or from IC_7_ to IC_12_): purified water was used as the first extraction solvent, followed by 50% aqueous methanol, then 50% aqueous ethanol, >99.9% methanol, and MeOH:EtOH (1/1, *v/v*), and >99.9% ethanol as the last extraction solvent.

Fungal biomass was separated from fermentation broth by centrifugation at 10,000 rpm for 10 min (Centrifuge Sigma 3 K 3OH) and vacuum filtration. Biomass was lyophilized (FreeZone 2.5 Liter 50C Benchtop freeze dryer, LABCONCO, Kansas City, MO, USA) and then weighed. The fungal pigments were extracted and fractionated from the mycelial cells of *Talaromyces* sp. 30570 using an alternative pressurized liquid extraction (PLE) process with eco-friendly solvents (water, methanol and ethanol) according to the method recently published by Lebeau et al. [25]. The PLE process was performed on a Dionex ASE system (ASE^TM^ 350 apparatus, Dionex, Germering, Germany). The weighed sample (lyophilized biomass) was transferred to a 10-mL stainless steel extraction cell equipped with two cellulose filters on the bottom and containing glass balls (diameter 0.25–0.50 mm). Then, the sample was subjected to a six-stage extraction procedure under high pressure as an attempt to entirely extract the intracellular pigments from the mycelium. The sequence of solvents was set to display a decreasing polarity profile: purified water was used as the first extraction solvent, followed by 50% methanol, then 50% ethanol, >99.9% methanol, and MeOH:EtOH (1/1, *v/v*), and >99.9% ethanol as the last extraction solvent (Figure 2). The PLE extraction conditions were: temperature: 90 °C, pressure >10 MPa, heating time: 5 min, static time: 18 min, flush: 100%, and purge: 5 min. Solvents (methanol and ethanol, 99.9%-HPLC quality) were obtained from Carlo Erba (Val de Reuil, France). Purified water was obtained from a Milli-Q system (EMD Millipore Co., Billerica, MA, USA).

The pigment content extracted from the mycelial biomass was analyzed by spectral analysis using a UV-visible spectrophotometer (UV-1800, Shimadzu Corporation, Tokyo, Japan) at 276 nm (i.e., λ_max_ of the well-known *Monascus*-like pigments rubropunctamin and monascurubramin) according the method earlier reported [25], and expressed in terms of milli-equivalents of polyketide-based pigments per liter of culture medium (i.e., volumetric production in meqv·L^−1^ in the culture medium). All experiments were conducted in triplicate. The extracts were then stored at 4 °C in an amber vial prior to chromatographic analysis.

### 2.3. HPLC-DAD Analysis

Pigment composition was characterized by reverse phase HPLC-DAD using the Ultimate 3000 apparatus (Dionex, Germering, Germany) based on the analytical method reported by Lebeau et al. [25]. Analytical conditions: 25 μL injection; Hypersil Gold^TM^ column (2.1 mm i.d. × 150 mm, 5 μm; Thermo Scientific Inc., Waltham, MA, USA); temperature 30 °C; elution with a water-acetonitrile-formic acid gradient system [25]. Data were analyzed by the Chromeleon v.6.80 software (Dionex). Acetonitrile (99.9%-HPLC quality) and formic acid (purity 99%) were obtained from Carlo Erba (Val de Reuil, France).

### 2.4. UHPLC-HR-ESI-MS Analyses

The pigments were identified by UHPLC- High Resolution Electrospray Ionization (HR-ESI) MS analyses according to the method published by Klitgaard et al. [29], and using the Agilent 1290 Infinity LC system with a DAD detector, coupled to an Agilent 6550 iFunnel Q-TOF with an electrospray ionization source (Agilent Technologies, Santa Clara, CA, USA), and a Poroshell 120 Phenyl–Hexyl column (2.1 mm i.d. × 250 mm, 2.7 μm; Agilent). The analytical conditions used in this study were those earlier reported by Klitgaard et al. [29]: the separation was performed at 60 °C with a water-acetonitrile gradient (with 20 mM formic acid) going from 10% (*v/v*) to 100% acetonitrile in 15 min, followed by 3 min with 100% acetonitrile. The flow rate was kept constant at 0.35 mL/min. Mass spectra were recorded as centroid data for *m/z* 85–1700 in positive and negative ESI-MS mode, with an acquisition rate of 10 spectra/s.

## 3. Results

### 3.1. Alternative Extraction and Characterization of Monascus-Like Azaphilone Pigments from the Marine-Derived Talaromyces sp. 30570 Strain

**Figure 3:**
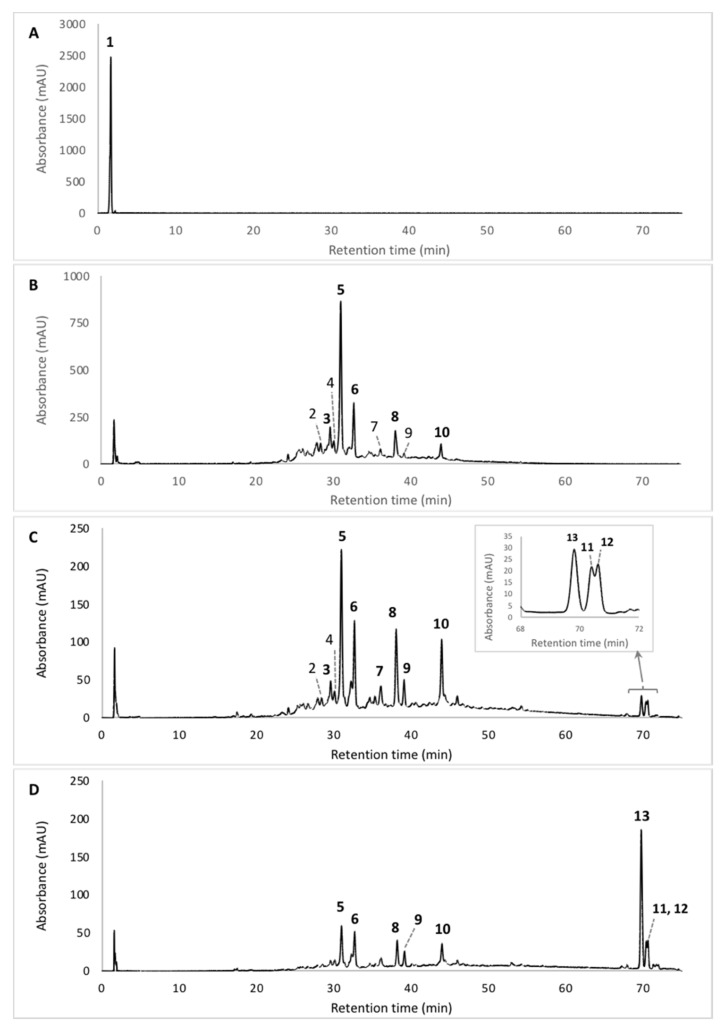
LC-DAD chromatograms of the fungal extracts from the mycelium of the marine-derived *Talaromyces* sp. 30570 strain cultivated in Potato Dextrose Broth (PDB). Pigments were extracted by pressurized liquid extraction: (**A**) water, (**B**) 50% aqueous methanol, (**C**) 50% aqueous ethanol, or (**D**) by pure methanol as extraction solvent. Assignment of the azaphilone red pigments **1**–**12** and ergosterol **13** were done by UV-visible and HRMS spectra. See Table 1 for the identification of the molecules.

Results from the analysis of the pigmented extracts using the alternative PLE method revealed a great diversity of the chemical structures of the *Talaromyces* sp. 30570 pigments. A series of intracellular extracts (IC) (Figure 2) were collected based on the PLE extraction procedure investigated, and their compositions in terms of fungal pigments were characterized by HPLC-DAD chromatography (Figure 3). Twelve pigmented compounds (compounds **1** to **12**) and one other colorless secondary metabolite (compound **13**, identified as ergosterol, see below) were identified. In particular, our results highlighted that the multi-step PLE procedure gives encouraging results in terms of selectivity of the extraction of the polyketide-based red pigments. This can be shown by two elements.

First, the initial extraction using hot pressurized water (90 °C and 10 MPa) enables the extraction with high selectivity of a highly polar pigment from fungal mycelium, namely compound **1**, found only in the aqueous fraction (Figure 3A), without co-extraction of side metabolites. Indeed, only one single peak (Rt 1.71 min; Figure 3A) was observed on the LC chromatogram. This compound **1** exhibits two absorption maxima at ca. 423 and 514 nm, which is characteristic of the *Monascus*-like nitrogen-containing azaphilone pigments [5,30]. Unfortunately, further dereplication experiments using HPLC-PDA-ESI/MS were not conclusive enough to fully elucidate the chemical structure of this highly polar red pigmented compound. Presumably, this highly polar compound **1**, exhibiting UV-visible λ_max_ at 201, 216, 244, 276, 423, and 514 nm, was presumed to be to a highly polar diglycoside derivative of a *Monascus*-like azaphilone red pigment. However, it cannot be concluded here that it represents only one compound, and alternative polar stationary phases (i.e., amide) should be used in further works for more relevant analysis of that/those compound(s).

Then, the following extractions using hot pressurized (90 °C and 10 MPa) hydroalcoholic mixtures such as 50% aqueous methanol (Figure 3B) or 50% aqueous ethanol (Figure 3C) enabled the extraction of others major *Monascus*-like red pigments from mycelium (pigments **2**–**12**) with a certain selectivity. Indeed, the extraction of other non-pigmented compounds such as ergosterol **13** (present in the intracellular metabolites produced by the mold) occurred only when less polar solvents, like pure methanol, were used (Figure 3D).

**Figure 4:**
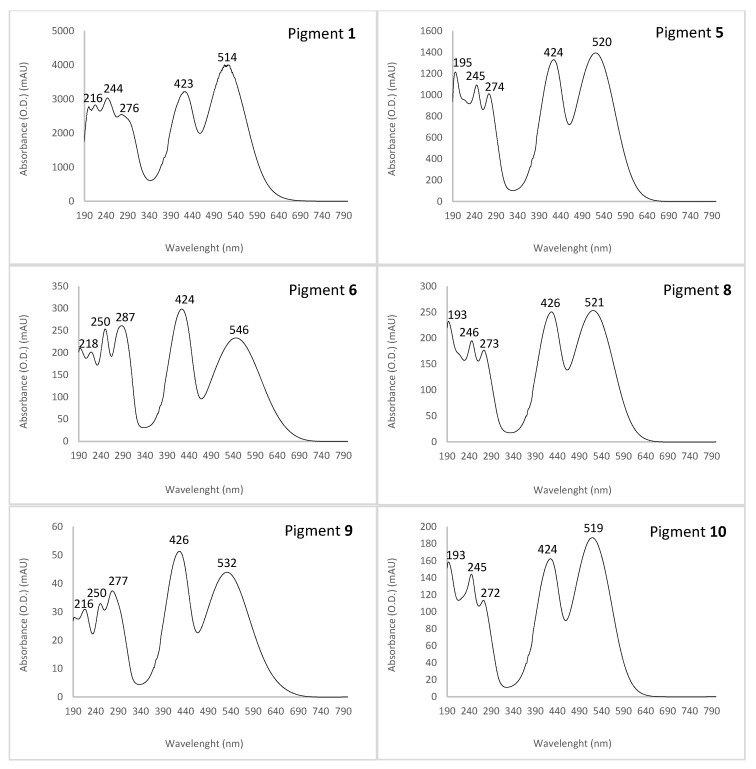
UV-visible absorption spectra of the major identified or assumed compounds **1**, **5**, **6**, **8**, **9** and **10** detected in intracellular extracts of the marine isolate *Talaromyces* sp. 30570 cultivated in PDB with reference to the chromatogram shown in Figure 3. Assignment of the nitrogen-containing azaphilone red pigments were done by UV-visible spectra and HRMS according to their mass to charge ratio. Pigments: 6-[(Z)-2-Carboxyvinyl]-N-GABA-PP-V **6**; *N*-threonine-monascorubramin **8**; *N*-glutaryl-rubropunctamin **9**; and monascorubramin **10**. Pigment **1**, not tentatively identified, was presumed to be to a highly polar compound, like a diglycoside derivative of a *Monascus*-like azaphilone red pigment. Then, it is presumed that the pigment **5** might reasonably be the molecule *N*-threonyl-rubropunctamin (or the acid form of the aforementioned PP-R), as recently reported by Rasmussen [30]. See Figure 5 for the chemical structure of the molecules.

The UV-visible absorption spectra of the major pigmented molecules (compounds **1**, **5**, **6**, **8**, **9** and **10** detected by HPLC-DAD, Figure 3) produced by the marine-derived *Talaromyces* sp. 30570 fungal strain are shown in Figure 4.

The UV-Vis absorption spectra gave some indications on the chemical structure of the pigments produced. All the compounds (**1**–**12**) responsible for the pigmentation (with absorption in visible region) shown the same UV-visible spectral characteristics, i.e., a mountain-like spectrum with three or four UV λ_max_ in the range 193–201, 216–218, 244–250 and 272–287 nm. Furthermore, all these aromatic compounds displayed the characteristic nitrogen-containing *Monascus*-like azaphilone red pigments double visible peaks around 430 and 515 nm (range 423–430 and 514–546 nm) (Figure 4, Table 1) in accordance with the literature data [5,30]. This unique chemical fingerprint of these pigments would suggest the presence of the monascorubramin or rubropunctamin-type chromophore in the molecule, as reported earlier [30].

**Figure 5:**
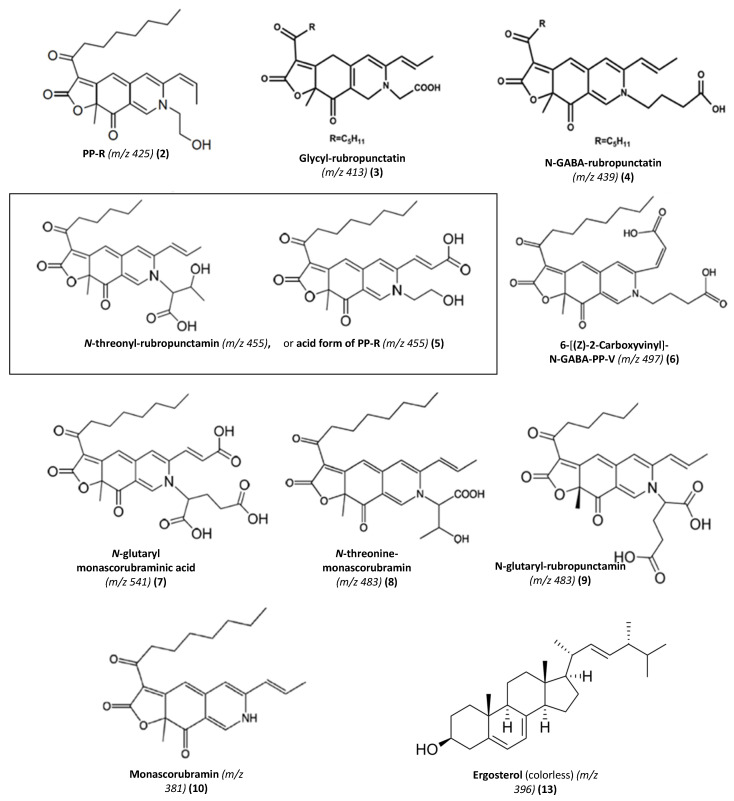
Main *Monascus*-like nitrogen-containing azaphilone pigments produced by the marine-derived *Talaromyces* sp. 30570 fungal strain. Assignment of the pigments (**2**–**10**) and ergosterol (**13**) were done by UV-visible spectra and HR-ESI-MS according to their mass to charge ratio. It is presumed that the major red pigment **5** produced by the fungal strain (see sidebar) might reasonably be the molecule *N*-threonyl-rubropunctamin (C_25_H_29_NO_7_, *m/z* 455) or the acid form (R-COOH) of the pigment PP-R (C_25_H_29_NO_7_, *m/z* 455) as recently reported by Rasmussen [30].

Thus, among the great number of compounds observed, twelve *Monascus*-type azaphilone red pigments (with absorption in the visible region due to the monascorubramin or rubropunctamin-type chromophore) were detected, and nine were tentatively identified as derivatives of nitrogen-containing azaphilone red pigment (see Figure 5 for the chemical structures of the major identified or assumed red pigments of *Talaromyces* sp. 30570). The retention time (Rt), UV-Vis λ_max_, accurate masses of parent ion and of adduct ions, color, molecular formula and average mass for each compound identified in the extracts of the marine-derived *Talaromyces* sp. 30570 strain cultivated in PDB are gathered in Table 1.

Among the derivatives of nitrogen-containing azaphilone red pigments identified, the compound **2** eluting at Rt. 28.52 min that presents UV-Vis λ_max_ at 192, 245, 274, 421 and 515 nm, was characteristic of the red pigment PP-R [7-(2-hydroxyethyl)-monascorubramin] which has previously been isolated from some other species of *Talaromyces* [14,15,16]. Indeed, the ACN-Na adduct ion [M + CAN + Na]^+^ observed at *m/z* 488.1820 was in agreement with the calculated masses of the C_25_H_31_NO_5_-CH_3_CN-Na^+^ adduct ion (*m/z* 488) and of molecular ion (*m/z* 425.22) to the red pigment PP-R suggesting a C_25_H_31_NO_5_ molecular formula (Table 1, Figure 5; Figure S1) [14,15,16].

Additionally, our results suggested that the compound **3** (Rt. 29.60 min; λ_max_ at 193, 245, 274, 421 and 518 nm; *m/z* 416.1960 [M + H]^+^) might reasonably correspond to the red pigment glycyl-rubropunctatin (C_23_H_27_NO_6_; average mass *m/z* 413.18) (Figure S2) previously isolated from *Monascus* cultures [31,32,33].

The UV-visible and HR-ESI-MS spectra of the compound **4** (Rt. 30.15 min; λ_max_ at 193, 245, 274, 426 and 515 nm) were characteristics to the red pigment *N*-GABA-rubropunctatin (GABA: γ-aminobutyric acid) [20]: the protonated molecular ion [M + H]^+^ observed at *m/z* 440.1936 was in agreement with the calculated mass of the *N*-GABA-rubropunctatin molecular ion (*m/z* 439.51) suggesting a C_25_H_29_NO_6_ molecular formula (Table 1, Figure 5; Figure S3) [20].

Interestingly, our results demonstrated that the major pigment produced by the marine-derived *Talaromyces* sp. 30570 strain, i.e., the compound **5** eluting at Rt. 30.97 min with UV-Vis λ_max_ at 195, 245, 274, 424 and 520 nm (Figure 3, Table 1) was a derivative of nitrogen-containing azaphilone red pigment and it is presumed that this pigment might reasonably be the molecule *N*-threonyl-rubropunctamin [25], or the acid form of the aforementioned PP-R, as recently reported by Rasmussen [30] from another species of *Talaromyces* (i.e., *T. atroroseus*). Indeed, this compound **5**, displayed a protonated molecular ion [M + H]^+^ at *m/z* 456.1543 (Figure S4), and the aforementioned derivatives *N*-threonyl-rubropunctamin and acid form of PP-R have the same nominal mass of 455.20, suggesting a C_25_H_29_NO_7_ molecular formula [25,30] (Table 1, Figure 5), which should be in agreement with the protonated molecular ion observed in this study. Further works are needed to purify and fully characterize this red pigment produced by the fungus by NMR analysis.

The compound **6** (Rt. 32.66 min; λ_max_ at 193, 218, 250, 287, 424 and 546 nm) was identified as the derivative 6-[(Z)-2-Carboxyvinyl]-*N*-GABA-PP-V. Its [M + H]^+^ ion, observed at *m/z* 498.1665, matched up well with the expected mass of the corresponding molecule (molecular ion *m/z* 497.54) suggesting a C_27_H_31_NO_8_ molecular formula (Table 1, Figure 5; Figure S5). This derivative of azaphilone red pigment has recently been isolated from another marine-derived strain of *Talaromyces* sp. 30548 (e.g., strain CBS 206.89 A, identified as *T. albobiverticillius*) also collected from the coral reef of the Réunion island [28,34].

The compound **7** (Rt. 36.11 min; λ_max_ at 196, 247, 288, 422 and 522 nm) was characteristic of the red pigment *N*-glutaryl-monascorubraminic acid (acid form) according to the data reported earlier [30]. Its protonated molecular ion [M + H]^+^ at *m/z* 542.1598 was consistent with the calculated mass of the molecular ion *m/z* 541.20 of the corresponding molecule, suggesting a C_28_H_31_NO_10_ molecular formula (Table 1, Figure 5; Figure S6) [30].

The compound **8** (Rt. 38.04 min; λ_max_ at 193, 246, 273, 426 and 521 nm) was characteristic to the red pigment *N*-threonine-monascorubramin [34]. Its protonated molecular ion [M + H]^+^ at *m/z* 484.1910 was in agreement with the calculated mass *m/z* 483.55 of the *N*-threonine-monascorubramin molecular ion, suggesting a C_27_H_33_NO_7_ molecular formula (Table 1, Figure 5; Figure S7) [34].

The compound **9** (Rt. 39.10 min; λ_max_ at 193, 216, 250, 277, 426 and 532 nm) was characteristic to the red pigment *N*-glutaryl-rubropunctamin. Its protonated molecular ion [M + H]^+^ at *m/z* 484.5110 supported by its ACN-Na adduct ion [M + ACN + Na]^+^ at *m/z* 546.1556 (Figure S8) coincided nicely with the expected mass 483.51 of the *N*-glutaryl-rubropunctamin (with formula C_26_H_29_NO_8_) isolated from other *Monascus* and *Talaromyces* species [15,34,35,36].

Then, the compound **10** (Rt. 43.95 min; λ_max_ at 193, 245, 272, 424 and 519 nm) seemed to correspond to the red pigment monascorubramin according to its protonated molecular ion *m/z* 381.1198 (Figure S9) relatively close to the calculated mass of the molecular ion *m/z* 381.19 of the corresponding molecule (C_23_H_27_NO_4_) [15,16].

**Figure 6:**
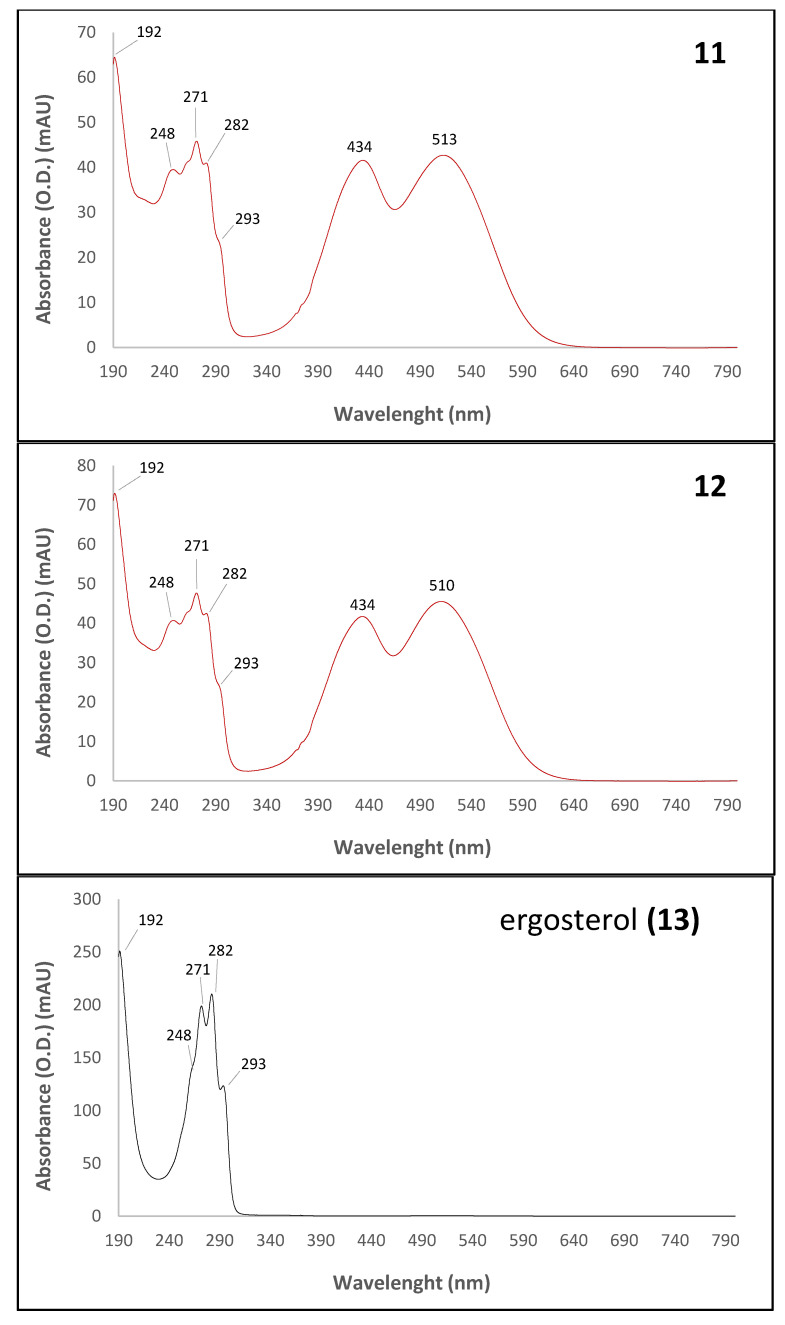
UV-visible spectra of the ergosterol (colorless compound **13**) and the two pigmented ergosterol derivatives of azaphilone compounds **11** and **12** with reference to the chromatograms shown in Figure 3, detected in the present study in intracellular extracts of the marine isolate *Talaromyces* sp. 30570.

In addition to these *Monascus*-like azaphilone pigments, no known mycotoxins were reported in the extracts obtained from the PLE extraction investigated here. Finally, our results suggested that the apolar and colorless compound **13** (Figure 6, Table 1) eluting at Rt. 69.78 min is assumed to be the molecule ergosterol (C_28_H_44_O; 396 g/mol), according to its similar absorption spectrum and to the HR-ESI-MS characteristic ion [M + H]^+^ at *m/z* 393.2693 (Figure S10). Indeed, ergosterol can undergo desaturation during LC-MS [37,38] (Table 1), consequently yielding a second molecular ion at *m/z* 393 in addition to the conventional molecular ion at *m/z* 397 [M + H]^+^. The results described in the present study are consistent with several earlier investigations which have highlighted the presence of ergosterol and derivatives of ergosterol from fungi [38]. On top of everything, ergosterol and its derivates are proven, with interesting bioactivities with potential uses in pharmaceutics [38,39].

Interestingly, based on the UV-visible spectra of the last molecules **11** and **12** (Figure 6), these two red pigments (with absorption at ca. 515 nm in the visible ‘red’ region) not tentatively identified by HPLC-PDA-ESI/MS (signal too weak) seemed to correspond to two pigmented ergosterol derivatives of azaphilone compounds. Indeed, they exhibited similar absorption spectra in the ultraviolet region to ergosterol molecule (i.e., λ_max_ at 248, 271, 282 and 293 nm). Surprisingly, they also displayed the characteristic nitrogen-containing *Monascus*-like azaphilone red pigments double visible peaks around 430 and 515 nm. To our knowledge, this is the first isolation of that kind of pigmented ergosterol derivatives of azaphilone red compounds from microorganisms. However, it was not possible to assign masses and chemical formulas to these two minor compounds.

### 3.2. Influence of the Nutrients Profile on the Production of Monascus-Like Azaphilone Red Pigments by the Marine-Derived Talaromyces sp. 30570 Strain

**Figure 7:**
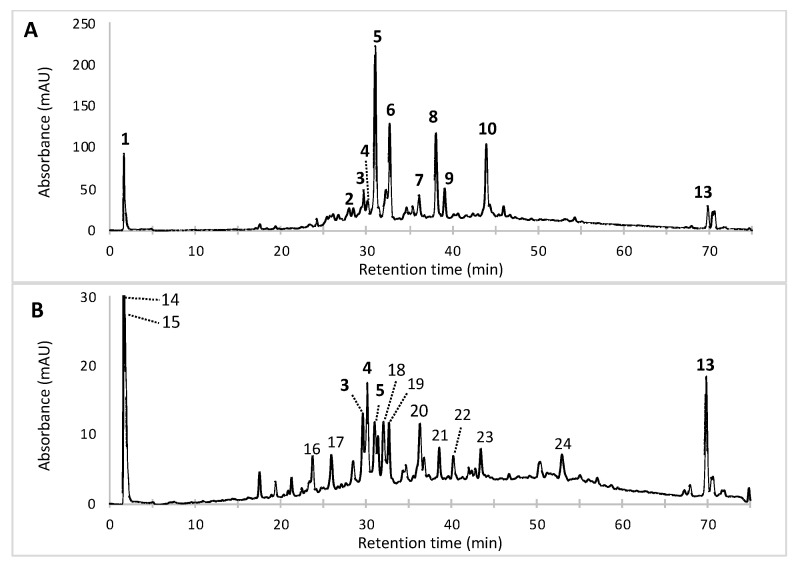
Chromatograms of the overall compounds detected by HPLC-DAD in the samples obtained by pressurized liquid extraction (PLE) using 50% aqueous ethanol as extraction solvent from the mycelium of the marine-derived *Talaromyces* sp. 30570 strain cultivated in submerged culture: (**A**) in Potato Dextrose Broth (PDB); and (**B**) in Defined Minimal Dextrose broth (DMD). Assignment of the polyketide-based compounds were done by UV-visible spectra and HRMS according to their mass to charge ratio. See Table 2 for the identification of the molecules.

Surprisingly, among the twelve red pigments detected in this study from the marine-derived *Talaromyces* sp. 30570 when cultivating in PDB, our results shown that only three well-known red pigments, i.e., the glycyl-rubropunctatin **3**, N-GABA-rubropunctatin **4** and the *N*-threonyl-rubropunctamin **5**, are common to both submerged culture conditions: in PDB (Figure 7A) and defined minimal dextrose broth (DMD) (Figure 7B). Our results, reported in Table 2, indicated that the nutrients’ profile of the fermentation broth has a clear impact on the pigment production by the marine-derived *Talaromyces* sp. 30570 fungal strain. These findings are corroborated by the results of earlier studies performed on *Talaromyces/Penicillium* species by Ogihara and Oishi [40] and Arai et al. [41], which have demonstrated that the fungal pigmentation will depend on the medium composition.

Indeed, in minimal nutrient condition (e.g., DMD broth) containing glucose and inorganic nitrogen source (with salts and bio-elements), the extraction and recovery of derivatives of nitrogen-containing azaphilone red pigments from the marine-derived *Talaromyces* sp. 30570 mycelia was very low. In this minimal nutrient condition, the fungus yielded compounds, which were mostly unpigmented (e.g., ergosterol **13** and the not identified compounds **14**–**16** and **20**–**22**) (Table 2). Thus, the yield of nitrogen-containing azaphilone red pigments in this minimal culture medium was very poor compared to PDB medium containing complex organic nitrogen (such as amino acids and proteins) and carbon sources. When cultivated in the minimal medium, the fungal strain produced only the glycyl-rubropunctatin **3**, the *N*-GABA-rubropunctatin **4**, and the *N*-threonyl-rubropunctamin **5** as red pigments (Figure 7, Table 2), whereas in PDB the fungus yielded twelve derivatives of nitrogen-containing azaphilone red pigments (pigments **1–12**; Table 1).

More particularly, it is worth noticing that the fungal strain was unable to produce monascorubramin **10** and derivatives of monascorubramin like *N*-glutaryl-monascorubraminic acid **7**, *N*-threonine-monascorubramin **8** and 6-[(Z)-2-Carboxyvinyl]-*N*-GABA-PP-V **6** when cultivated in minimal nutrient condition without amino acids and proteins in the medium. These findings are consistent with the results described in previous reports, suggesting that organic sources of nitrogen favor high red pigment production by *Talaromyces/Penicillium* species [19]. Thus, the non-production of monascorubramin and its amino derivates, when the strain of *Talaromyces* sp. 30570 was cultivated in minimal medium could be explained by the unavailability of more suitable organic nitrogen sources (amino acid, peptides, etc.) for the biosynthesis of such nitrogen-based azaphilone compounds. This suggests that the presence of amino acids and peptides in PDB medium enables the functionality of this specific pathway.

## 4. Discussion

### 4.1. Efficiency and Selectivity of the Alternative Pressurized Liquid Extraction (PLE) of Azaphilone Red Pigments from the Mycelial Cells of the Marine-Derived Talaromyces sp. 30570

The *Monascus*-like azaphilone red pigments are water-soluble, thus they are readily extracted with polar solvents [42]. Our results revealed the alternative PLE technique to be highly efficient in removing *Monascus*-like azaphilone red pigments from mycelial biomass of the marine-derived *Talaromyces* sp. 30570 by using water, methanol and/or ethanol at 90 °C and 10 MPa as extraction solvents. The sole use of these eco-friendly solvents, which can be biosourced, adds to the novelty of our results. Indeed, solvents such as ethanol and methanol can be produced from carbon-neutral homoacetogenic gas fermentation [43] and biogas produced from wastes, respectively, strengthening the sustainability of such process. This alternative PLE technique should be considered as a promising eco-friendly extraction process for natural products from biological samples [5,24,25,26,39,44]. It also opens the way to further optimizations to the solvent mixture to use for isolating specific polyketide red pigments. Additionally, the use of pressurized nitrogen gas protects the target molecules (fungal pigments) from oxidation and ensures a higher quality of the recovered target molecules.

Although the different azaphilone red pigments identified isolated in this study have already been previously individually isolated in some species from *Monascus* (e.g., *M. ruber)* [20,21] and *Talaromyces (*e.g., *T. atroroseus* [30] and *T. albobiverticillius* [34]), this is the first report to our knowledge of the concomitant occurrence of these twelve azaphilone pigments in a fungal extract obtained from a culture of a wildtype marine strain of *Talaromyces* (e.g., *Talaromyces* sp. 30570). Azaphilonoids, and in particular, derivates of the pigmented monascorubramin and rubropunctamin produced by non-toxicogenic species from *Talaromyces* sp., are non-toxic compounds highly wanted in pharmaceutical industries due to their bioactivities (antibiotic, anti-inflammatory activities amongst others) [19,45,46]. Therefore, this ability to produce molecules with high industrial interest by the wildtype marine strain of *Talaromyces* sp. 30570 could be further expended and scaled up to commercial production. It is worthy of notice that other studies performed on strains of *T. atroroseus* [30] and *T. alboverticillius* [34] have reported the presence, in fungal extracts, of different pigmented compounds, such as monascusone A, monascorubrin, PP-V, PP-Y, PP-O, as well as new pigmented azaphilone-like molecules, formerly known as atrorosins [47,48], and not detected in fungal extracts of *Talaromyces* sp. 30570 studied here. This observation clearly highlights the vast diversity of the polyketide-based pigments biosynthesized by the *Talaromyces* species, and in particular those from marine origin. This could also suggest the occurrence of an intraspecies diversity, as is already the case in other complexes of mold species, like in the *Talaromyces pinophilus* species complex [49] and in the *Fusarium oxysporum* species complex [39].

### 4.2. Putative Metabolic Pathway for the Production of Derivatives of Nitrogen-Containing Monascus-Like Azaphilone Red Pigments in the Marine-Derived Talaromyces sp. 30570

*Monascus*-like azaphilone pigments are colored metabolites with a pyrone–quinone structure [5]. They involve biosynthetic pathways catalyzed by multiple polyketide synthase enzymes (PKS). For over a decade now, a number of studies have attempted to assess the biosynthetic pathways of *Monascus*-like azaphilone pigments in the genera of *Monascus* and *Talaromyces/Penicillium* [17,18,19,20,21]. In the in vitro study of Chen et al. [20], the metabolic pathway of *Monascus*-like azaphilone pigments was elucidated in *Monascus ruber* M7. Then, Chen et al. [21] demonstrated that the biosynthetic gene clusters responsible for the pigment production in these fungi share orthologous genes for a conserved unitary trunk pathway [21]. They also described four physiological strategies responsible for the diversity of the *Monascus*-like azaphilone pigment structures in *Monascus* and *Talaromyces/Penicillium* genera, as well as differences regarding the gene organization, copy numbers and allelic diversity [20,21,22]. They mentioned that five and four gene clusters have been described to date from the *Monascus* and *Talaromyces* genera, respectively [20,21].

**Figure 8:**
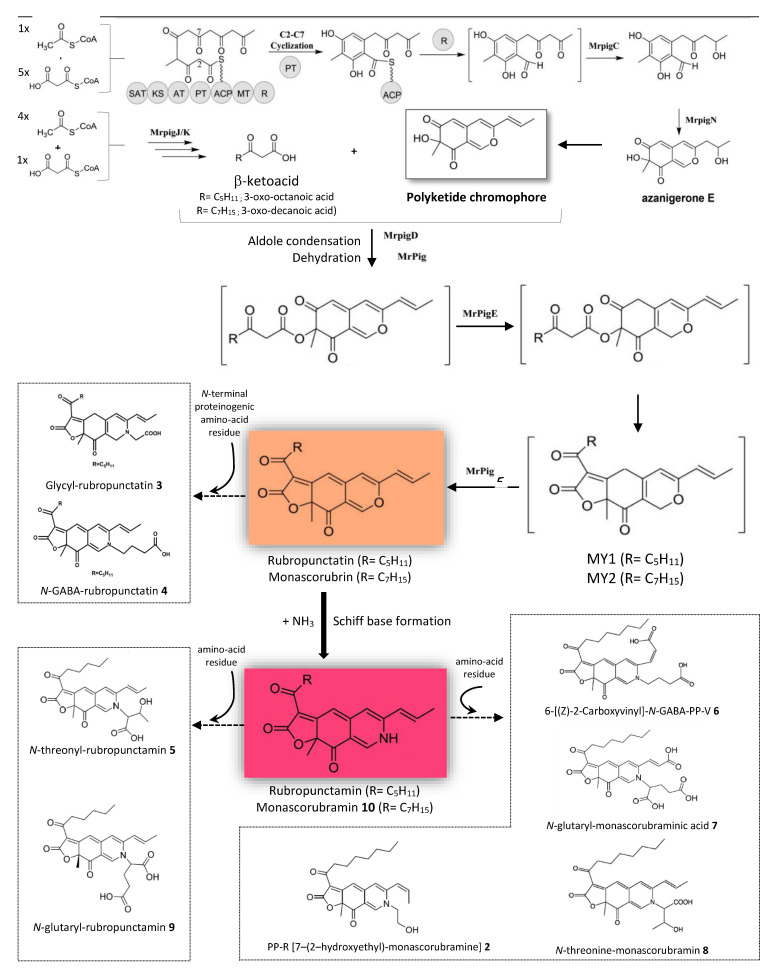
Putative metabolic pathway for the production of derivatives of nitrogen-containing azaphilone red pigments in the marine-derived *Talaromyces* sp. 30570 strain, based on the previously reported models for *Monascus*-like azaphilone pigments biosynthesis in other *Monascus* and *Talaromyces/Penicillium* species according to Chen et al. [20,21] and reviewed by Liu et al. [22]. Names of the enzymes are specified with reference to those identified in *M. ruber* M7 [20,21]. The non-reducing polyketide synthase *MrPigA* gene cluster encodes for a starter unit acyl carrier protein transacylase (SAT), a ketoacyl synthase (KS), an acyltransferase (AT), a product template (PT), two acyl carrier proteins (ACP), a *C*-methyltransferase (MT) and a reductive release domain (R). *MrPigC*: C-11-Ketoreductase; *MrPigD*: 4-*O*-Acyltransferase; *MrPigM*: *O*-Acyltransferase; *MrPigN*: FAD-dependent monooxygenase; *MrPigE*: NAD(P)H-dependent oxidoreductase; *MrPigF*: FAD-dependent oxidoreductase; *MrPigO*: Deacetylase. This figure was adapted from Chen et al. [20] and Liu et al. [22] with some modifications.

Thus, based on the previously reported models for *Monascus*-like azaphilone pigments biosynthesis in other *Monascus* and *Talaromyces/Penicillium* species [20,21,22], a putative pathway for the biosynthesis of derivatives of nitrogen-containing *Monascus*-like azaphilone red pigments and intermediates thereof in this marine-derived *Talaromyces* sp. 30570 strain was proposed in this study and described in Figure 8. Concerning the trunk pathway, the biosynthesis of yellow and orange azaphilone pigments is initiated via the polyketide pathway by a nonreducing PKS (known as *MrPigA* in *M. ruber* M7) [20,21] that features different domains like a starter unit acyl carrier protein transacylase (SAT), a ketoacyl synthase (KS), an acyltransferase (AT), a product template (PT), two acyl carrier proteins (ACP), a *C*-methyltransferase (MT) and a reductive release domain (R) [20,21,22] as shown in Figure 8. Then, the first stable azaphilone pigments intermediate (FK17-P2a) was synthetized by a ketoreductase (e.g., *MrPigC* in *M. ruber* M7) [20,21]. Next, an FAD-dependent monooxygenase (e.g., *MrpigN* in *M. ruber* M7) [20,21] is then critical to obtain the bicyclic pyran-containing azaphilone core. The polyketide-chromophore may come from further modifications to this azaphilone core by enzymatic or non-enzymatic reaction. Finally, the orange azaphilone pigments are formed by the esterification of a *β*-ketoacid (e.g., 3-oxo-octanoic acid and 3-oxo-decanoic acid resulted from the fatty acids biosynthetic pathway by a dedicated two-subunit fatty acid synthetase: e.g., MrPigJ/K in *M. ruber* M7) to the aforementioned polyketide-based chromophore, as shown in Figure 8.

Woo et al. [17] also described similar findings in *P. marneffei* PM1: they demonstrated that the biosynthetic pathway for the production of azaphilone pigments is regulated by a gene cluster (*pks3*) that also encodes for KS, AT, ACP, MT and thiolester reductase (R) domains [17]. These authors suggested that the synthesis of the orange *Monascus*-like azaphilone pigments such as rubropunctatin and monascorubrin begins through the polyketide pathway, initially modulated by this *pks3* gene cluster in *P. marneffei* PM1 [17]. These orange pigments and their well-known derivatives, e.g., the pigments glycyl-rubropunctatin **3** and *N*-GABA-rubropunctatin **4** isolated in this study, may be formed by the esterification of 3-oxo-octanoic acid or 3-oxo-decanoic acid to the polyketide-based chromophore [17]. From there, red derivatives of *Monascus*-like azaphilone pigments can be synthetized by Schiff base formation reactions [17,18,19,22]. Indeed, the amination of the orange *Monascus*-like azaphilone pigments with proteins, amino acids or nucleic acids yields the azaphilone red pigments, including the derivatives of rubropunctamin (e.g., *N*-threonyl-rubropunctamin **5** and *N*-glutaryl-rubropunctamin **9**) and the derivatives of monascorubramin **10**, like PP-R **2**, 6-[(Z)-2-Carboxyvinyl]-*N*-GABA-PP-V **6**, *N*-glutaryl-monascorubraminic acid **7** and *N*-threonine-monascorubramin **8** identified in this study from the marine-derived *Talaromyces* sp. 30570 strain (Figure 8).

## 5. Conclusions

In this study, we demonstrated the potential of the marine-derived fungal strain *Talaromyces* sp. 30570 to produce a wide variety of water-soluble *Monascus*-like azaphilone red pigments with respect to the medium composition. Such environment-dependent responses confirmed that the manipulation of the culture conditions (in particular, the presence of organic nitrogen sources) may trigger the expression of certain biosynthetic pathways and the production of a high amount of nitrogen-containing red azaphilone pigments by the fungi. Among the twelve different pigments detected in the fungal extract, nine derivatives of nitrogen-containing azaphilone red pigments were identified. *N*-threonyl-rubropunctamin or the acid form of the pigment PP-R, 6-[(Z)-2-Carboxyvinyl]-*N*-GABA-PP-V, *N*-threonine-monascorubramin, *N*-glutaryl-rubropunctamin, and monascorubramin were the major pigmented compounds. Bioproduction of these molecules occurred only when complex organic nitrogen sources were present in the culture medium. These findings are important for the field of the selective production of these fungal red azaphilones. They may represent relevant metabolites for the industries. Indeed, among the natural colorants, the red ones are the most interesting, as they are increasingly used in human foods and in ingested drugs. These fungal red azaphilones are not only ‘colored,’ they often exhibit remarkable antibiotic and antitumoral activities, and are of interest due to their applications in the design of new pharmaceutical products.

## Figures and Tables

**Table 1:** Overall compounds (12 derivatives of nitrogen-containing azaphilone red pigments and ergosterol **13**) identified from the fungal extracts of the marine-derived *Talaromyces* sp. 30570 fungal strain cultivated in Potato Dextrose Broth (PDB), with reference to the chromatograms shown in Figure 3.

No	Rt. (min)	UV-Vis λ_max_ (nm)	Observed Peak HR-ESI MS *(m/z)*	Tentative Identification (Identified or Assumed Compounds)	Proposed Molecular Formula	Monoisotopic Mass in Da *(Mass Error)* ^(1)^	Ref.
**1**	1.71	201, 216, 244, 276, 423, 514	n.d.	Diglycoside derivative of a *Monascus*-like azaphilone red pigment (n.i.)	n.d.	n.d.	-
**2**	28.52	192, 245, 274, 421, 515	488.1820 [M + CAN + Na]^+^	**PP-R** [7-(2-hydroxyethyl)-monascorubramin]	C_25_H_31_NO_5_	425.22 *(0.0380)*	[14,15,16]
**3**	29.60	193, 245, 274, 421, 518	416.1960 [M + H]^+^	**Glycyl-rubropunctatin**	C_23_H_27_NO_6_	413.18 *(2.0160)*	[31,32,33]
**4**	30.15	193, 245, 274, 426, 515	440.1936 [M + H]^+^	***N*-GABA-rubropunctatin** (GABA: γ-aminobutyric acid)	C_25_H_29_NO_6_	439.51 *(0.3164)*	[20]
**5**	30.97	195, 245, 274, 424, 520	456.1543 [M + H]^+^	***N*-threonyl-rubropunctamin** (or **acid form of PP-R**) (presumed)	C_25_H_29_N0_7_	455.20 *(0.0457)*	[25,30]
**6**	32.66	193, 218, 250, 287, 424, 546	498.1665 [M + H]^+^	**6-[(Z)-2-Carboxyvinyl]-*N*-GABA-PP-V**	C_27_H_31_N0_8_	497.54 *(0.3735)*	[28,34]
**7**	36.11	196, 247, 288, 422, 522	542.1598 [M + H]^+^	***N*-glutaryl-monascorubraminic acid**	C_28_H_31_N0_10_	541.20 *(0.038)*	[30]
**8**	38.04	193, 246, 273, 426, 521	484.1910 [M+H]^+^	***N*-threonine-monascorubramin**	C_27_H_33_N0_7_	483.55 *(0.0402)*	[34]
**9**	39.10	193, 216, 250, 277, 426, 532	484.5110 [M + H]^+^ 546.1556 [M + CAN + Na]^+^	***N*-glutaryl-rubropunctamin**	C_26_H_29_N0_8_	483.51 *(0.0010)*	[15,34,35,36]
**10**	43.95	193, 245, 272, 424, 519	381.1198 [M + H]^+^	**Monascorubramin**	C_23_H_27_NO_4_	381.19 *(1.0702)*	[15,16]
**11**	70.40	192, 248, 271, 282, 293, 434, 513	n.d.	Derivative of a *Monascus*-like azaphilone red pigment (n.i.)	n.d.	n.d	-
**12**	70.64	192, 248, 271, 282, 293, 434, 510	n.d.	Derivative of a *Monascus*-like azaphilone red pigment (n.i.)	n.d.	n.d.	-
**13**	69.78	192, 248, 271, 282, 293	393.2693	**Ergosterol** (colorless compound)	C_28_H_44_O	396.65 *(0.3807)*	[37,38]

n.d.: not determined.; n.i.: not identified; ^(1)^ the mass error (Da) between the observed MS peaks and proposed formula (for the molecular ion).

**Table 2:** Overall compounds identified from the fungal extracts of the marine-derived *Talaromyces* sp. 30570 fungal strain cultivated in 2 different submerged cultures: in Potato Dextrose Broth (PDB) and in Defined Minimal Dextrose broth (DMD), with reference to the chromatograms shown in Figure 7.

No	Rt. (min)	UV-Vis λ_max_ (nm) (Bold: λ_max_ in Visible)	Tentative Identification (Identified or Assumed Compounds)	Polyketide-Based Compounds Content (meqv.L^−1^)	
				in PDB ^(1)^	in DMD ^(1)^
**1**	1.71	201, 216, 244, 276, **423, 514**	**Diglycoside derivative of a *Monascus*-like azaphilone red pigment**	124.8 ± 5.0	-
**2**	28.52	192, 245, 274, **421, 515**	**PP-R** [7-(2-hydroxyethyl)-monascorubramin]	6.7 ± 0.4	-
**3**	29.60	193, 245, 274, **421, 518**	**Glycyl-rubropunctatin**	22.1 ± 1.3	7.3 ± 0.3
**4**	30.15	193, 245, 274, **426, 515**	***N*-GABA-rubropunctatin** (GABA: γ-aminobutyric acid)	8.0 ± 0.3	4.9 ± 0.4
**5**	30.97	195, 245, 274, **424, 520**	***N*-threonyl-rubropunctamin** (or **acid form of PP-R**)	83.4 ± 4.1	9.0 ± 0.8
**6**	32.66	193, 218, 250, 287, **424, 546**	**6-[(Z)-2-Carboxyvinyl]-*N*-GABA-PP-V**	33.5 ± 1.3	-
**7**	36.11	196, 247, 288, **422, 522**	***N*-glutaryl-monascorubraminic acid**	7.3 ± 0.4	-
**8**	38.04	193, 246, 273, 426, 521	***N*-threonine-monascorubramin**	24.3 ± 0.6	-
**9**	39.10	193, 216, 250, 277, **426, 532**	***N*-glutaryl-rubropunctamin**	5.7 ± 0.2	-
**10**	43.95	193, 245, 272, **424, 519**	**Monascorubramin**	19.6 ± 0.9	-
**11**	70.40	192, 248, 271, 282, 293, **434, 513**	**Derivative of a *Monascus*-like azaphilone red pigment** (n.i.)	4.1 ± 0.2	-
**12**	70.64	192, 248, 271, 282, 293, **434, 510**	**Derivative of a *Monascus*-like azaphilone red pigment** (n.i.)	4.2 ± 0.4	-
**13**	69.78	192, 248, 271, 282, 293	**Ergosterol** (colorless compound)	24.0 ± 1.0	73.8 ± 1.9
**14**	1.63	198, 260	Colorless compound (n.i.)	-	31.1 ± 1.2
**15**	2.01	196, 258	Colorless compound (n.i.)	-	6.1 ± 0.3
**16**	23.79	203, 256, 298	Colorless compound (n.i.)	-	6.3 ± 0.3
**17**	25.93	196, 264, 278, **479**	Yellow pigment (n.i.)	-	5.2 ± 0.2
**18**	32.03	193, 252, 294, **428, 546**	Purple-red pigment (n.i.)	-	5.0 ± 0.3
**19**	32.73	192, 220, 246, 289, **415, 546**	Purple-red pigment (n.i.)	-	3.8 ± 0.3
**20**	36.30	193, 260, 274	Colorless compound (n.i.)	-	2.7 ± 0.2
**21**	38.57	192, 211, 243, 391	Colorless compound (n.i.)	-	1.5 ± 0.2
**22**	40.21	210, 292, 421	Colorless compound (n.i.)	-	0.4 ± 0.1
**23**	43.43	192, 280, **409, 431**	Yellow pigment (n.i.)	-	0.4 ± 0.1
**24**	52.93	192, 248, 271, 282, 293, **414**	Yellow pigment (n.i.)	-	17.4 ± 1.1

^(1)^ mean (± standard deviation) expressed in meqv.L^-1^ of polyketide-based compounds produced in PDB and DMD broths.

## Supplementary Materials

The following are available online at https://www.mdpi.com/2076-2607/8/12/1920/s1, Figure S1. Mass spectra of the assumed compound PP-R **2**; Figure S2. Mass spectra of the assumed compound Glycyl-rubropunctatin **3**; Figure S3. Mass spectra of the assumed compound *N*-GABA-rubropunctatin **4**; Figure S4. Mass spectra of the assumed compound *N*-threonyl-rubroptunctamin or acid form of PP-R **5**; Figure S5. Mass spectra of the assumed compound 6-[(Z)-2-Carboxylvinyl]-*N*-GABA-PP-V **6**; Figure S6. Mass spectra of the assumed compound *N*-glutaryl-monascorubraminic acid **7**; Figure S7. Mass spectra of the assumed compound *N*-threonine-monascorubramin **8**; Figure S8. Mass spectra of the assumed compound *N*-glutaryl-rubropunctamin **9**; Figure S9. Mass spectra of the assumed compound Monascorubramin **10**; Figure S10. Mass spectra of the assumed compound Ergosterol **13**.
