# Supplementary material for: Alternative Extraction and Characterization of Nitrogen-Containing Azaphilone Red Pigments and Ergosterol Derivatives from the Marine-Derived Fungal Talaromyces sp. 30570 Strain with Industrial Relevance
Source: Microorganisms. 2020 Dec 3;8(12):1920. doi: 10.3390/microorganisms8121920 (PMC7761761; doi:10.3390/microorganisms8121920)
Supplement: Supplementary file 1 [file microorganisms-08-01920-s001.pdf]

microorganisms

MDPI

Article (SI Special Issue "Microbial Secondary Metabolites and Biotechnology")

Alternative extraction and characterization of nitrogen-containing azaphilone red pigments and ergosterol derivatives from the marine-derived fungal *Talaromyces* sp 30570 strain with industrial relevance

Juliana Lebeau [1], Thomas Petit [1,2], Mireille Fouillaud [1], Laurent Dufossé [1] and Yanis Caro [1,2,*]

[1] Laboratoire de Chimie et de Biotechnologie des Produits Naturels, CHEMBIOPRO, Université de La Réunion, 15 avenue René Cassin, CS 92003, F-97744, Saint-Denis, Réunion, France; juliana.lebeau@univ-reunion.fr; thomas.petit@univ-reunion.fr; mireille.fouillaud@univ-reunion.fr; laurent.dufosse@univ-reunion.fr; yanis.caro@univ-reunion.fr

[2] Département Hygiène Sécurité Environnement (HSE), IUT La Réunion, Université de La Réunion, 40 avenue de Soweto, BP 373, F-97455, Saint-Pierre, Réunion, France; ; thomas.petit@univ-reunion.fr; yanis.caro@univ-reunion.fr

* Correspondence: yanis.caro@univ-reunion.fr (Y.C.)

Received: date; Accepted: date; Published: date

Abstract: Many species of *Talaromyces* of marine origin could be considered as non-toxigenic fungal cell factory. Some strains could produce water-soluble active biopigments in submerged cultures. These fungal pigments are of interest due to their applications in the design of new pharmaceutical products. In this study, the azaphilone red pigments and ergosterol derivatives produced by a wild type of *Talaromyces* sp 30570 (CBS 206.89 B) marine-derived fungal strain with industrial relevance were described. The strain was isolated from the coral reef of the Réunion island. An alternative extraction of the fungal pigments using high-pressure with eco-friendly solvents was studied. Twelve different red pigments were detected including two pigmented ergosterol derivatives. Nine metabolites were identified using HPLC-PDA-ESI/MS as *Monascus*-like azaphilone pigments. In particular, derivatives of nitrogen-containing azaphilone red pigment, like PP-R, 6-[(Z)-2-Carboxyvinyl]-N-GABA-PP-V, *N*-threonine-monascorubramin, *N*-glutaryl-rubropunctamin, monascorubramin, and presumed *N*-threonyl-rubropunctamin (or acid form of the pigment PP-R) were the major pigmented compounds produced. Interestingly, the bioproduction of these red pigments occurred only when complex organic nitrogen sources were present in the culture medium. These findings are important for the field of the selective production of *Monascus*-like azaphilone red pigments for the industries.

Keywords: *Talaromyces*; azaphilone; marine fungi; *N*-threonyl-rubropunctamin; PP-R; greener extraction; red pigments; fungal pigments.

Supplementary Materials

microorganisms

Supplementary Materials

Figure S1. Mass spectra of the assumed compound PP-R **2** detected in the present study in intracellular extracts of the marine isolate *Talaromyces* sp. 30570

Figure S2. Mass spectra of the assumed compound Glycyl-rubropunctatin **3** detected in the present study in intracellular extracts of the marine isolate *Talaromyces* sp. 30570

Figure S3. Mass spectra of the assumed compound N-GABA-rubropunctatin **4** detected in the present study in intracellular extracts of the marine isolate *Talaromyces* sp. 30570

Figure S4. Mass spectra of the assumed compound *N*-threonyl-rubroptunctamin or acid form of PP-R **5** detected in the present study in intracellular extracts of the marine isolate *Talaromyces* sp. 30570

Figure S5. Mass spectra of the assumed compound 6-[(Z)-2-Carboxylvinyl]-N-GABA-PP-V **6** detected in the present study in intracellular extracts of the marine isolate *Talaromyces* sp. 30570

Figure S6. Mass spectra of the assumed compound *N*-glutaryl-monascorubraminic acid **7** detected in the present study in intracellular extracts of the marine isolate *Talaromyces* sp. 30570

Figure S7. Mass spectra of the assumed compound *N*-threonine-monascorubramin **8** detected in the present study in intracellular extracts of the marine isolate *Talaromyces* sp. 30570

Figure S8. Mass spectra of the assumed compound *N*-glutaryl-rubropunctamin **9** detected in the present study in intracellular extracts of the marine isolate *Talaromyces* sp. 30570

Figure S9. Mass spectra of the assumed compound Monascorubramin **10** detected in the present study in intracellular extracts of the marine isolate *Talaromyces* sp. 30570

Figure S10. Mass spectra of the assumed compound Ergosterol **13** detected in the present study in intracellular extracts of the marine isolate *Talaromyces* sp. 30570

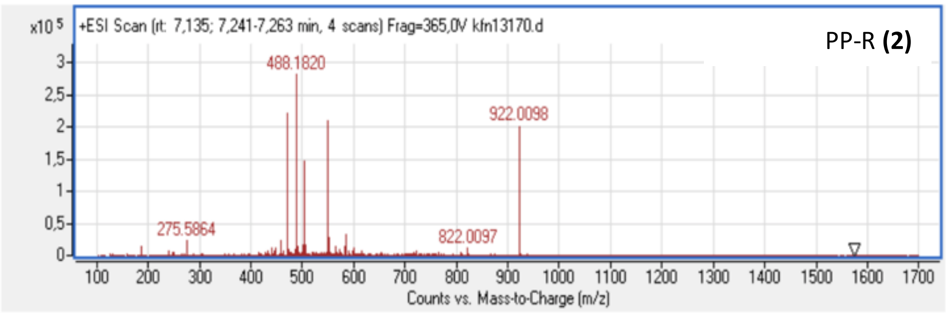

Figure S1. Mass spectra of the assumed compound PP-R **2** detected in the present study in intracellular extracts of the marine isolate *Talaromyces* sp. 30570

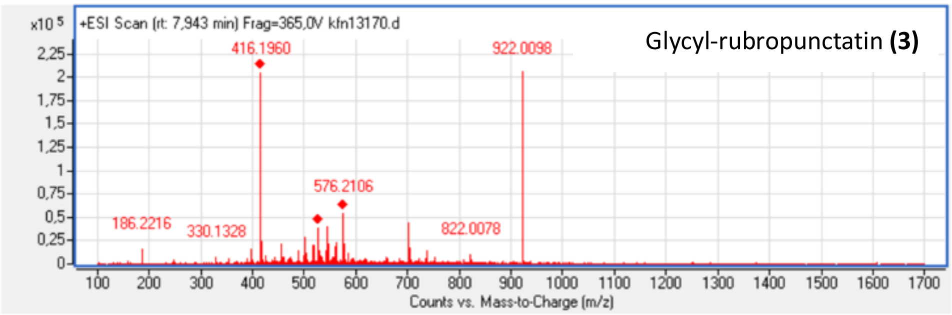

Figure S2. Mass spectra of the assumed compound Glycyl-rubropunctatin **3** detected in the present study in intracellular extracts of the marine isolate *Talaromyces* sp. 30570

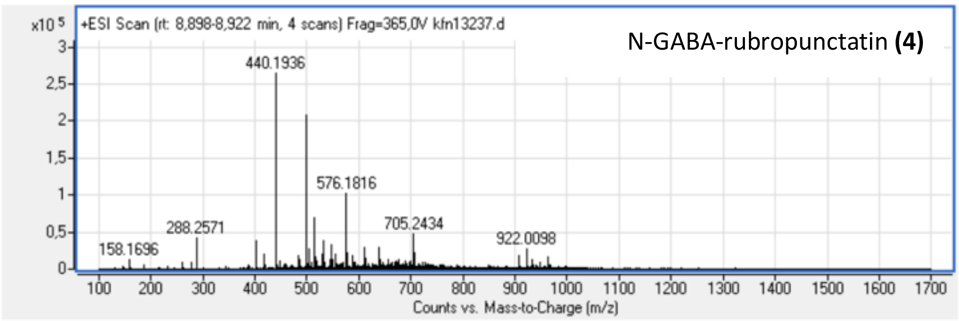

Figure S3. Mass spectra of the assumed compound N-GABA-rubropunctatin **4** detected in the present study in intracellular extracts of the marine isolate *Talaromyces* sp. 30570

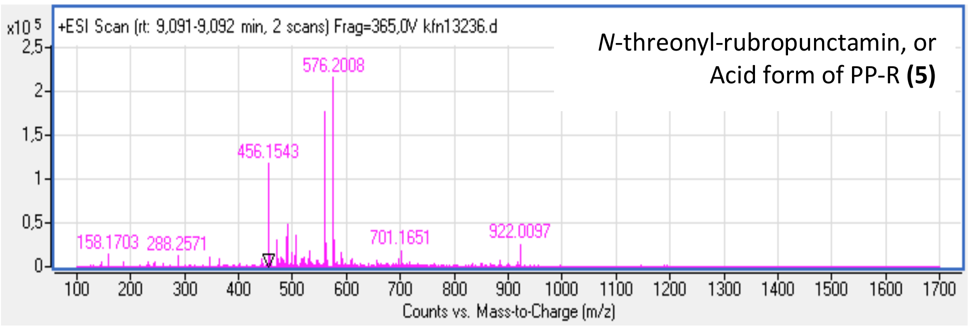

Figure S4. Mass spectra of the assumed compound *N*-threonyl-rubroptunctamin or acid form of PP-R **5** detected in the present study in intracellular extracts of the marine isolate *Talaromyces* sp. 30570

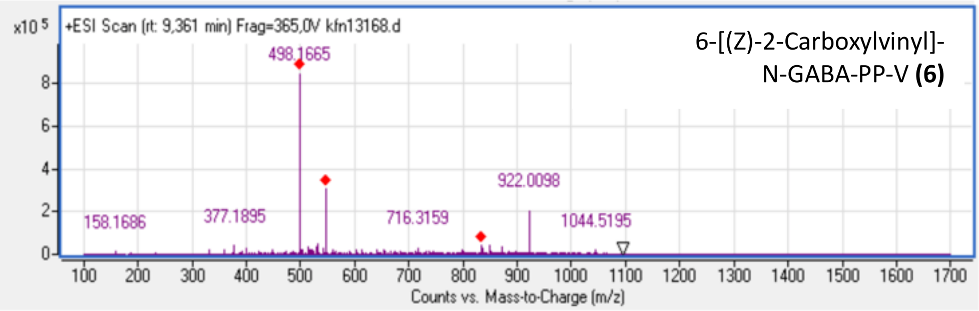

Figure S5. Mass spectra of the assumed compound 6-[(Z)-2-Carboxylvinyl]-N-GABA-PP-V **6** detected in the present study in intracellular extracts of the marine isolate *Talaromyces* sp. 30570

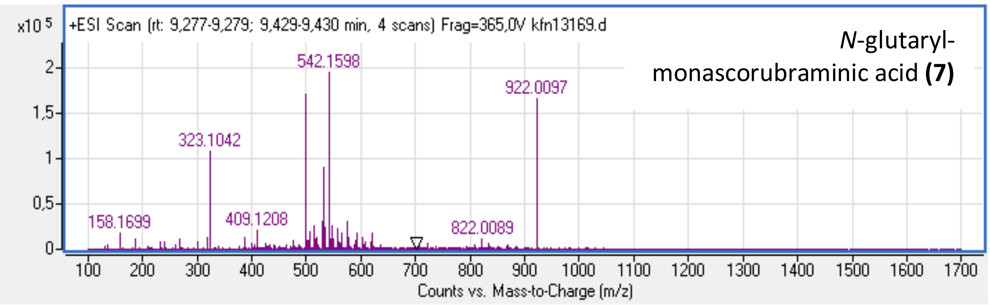

Figure S6. Mass spectra of the assumed compound *N*-glutaryl-monascorubraminic acid **7** detected in the present study in intracellular extracts of the marine isolate *Talaromyces* sp. 30570

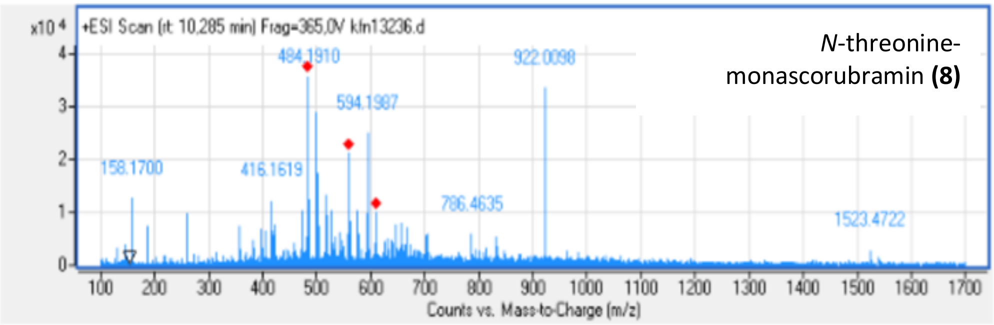

Figure S7. Mass spectra of the assumed compound *N*-threonine-monascorubramin **8** detected in the present study in intracellular extracts of the marine isolate *Talaromyces* sp. 30570

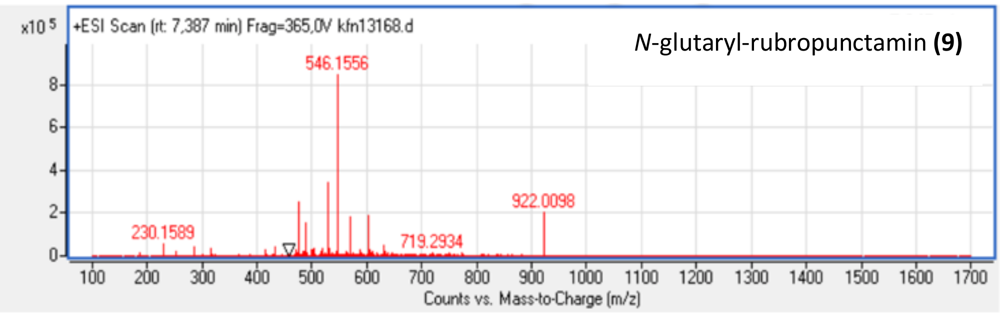

Figure S8. Mass spectra of the assumed compound *N*-glutaryl-rubropunctamin **9** detected in the present study in intracellular extracts of the marine isolate *Talaromyces* sp. 30570

microorganisms

MDPI

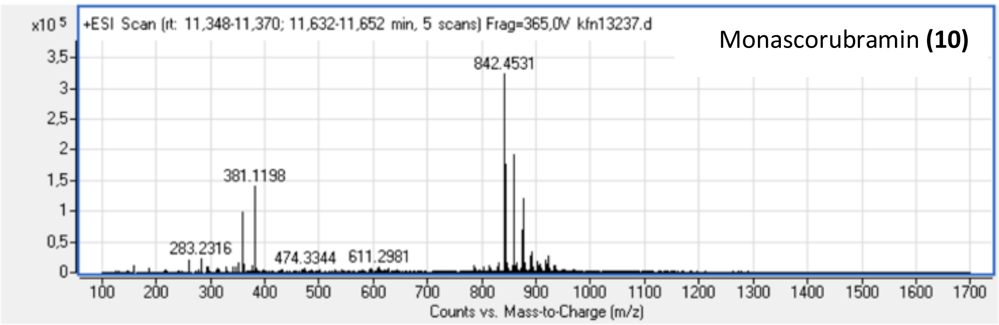

Figure S9. Mass spectra of the assumed compound Monascorubramin **10** detected in the present study in intracellular extracts of the marine isolate *Talaromyces* sp. 30570

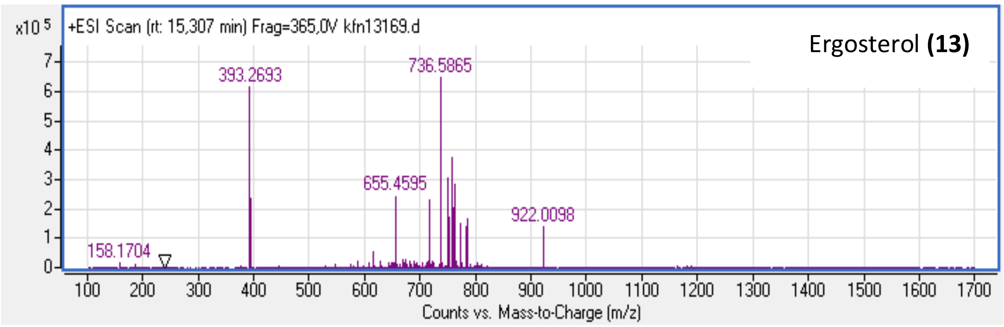

Figure S10. Mass spectra of the assumed compound Ergosterol **13** detected in the present study in intracellular extracts of the marine isolate *Talaromyces* sp. 30570
